# Interpretable deep learning survival predictive tool for small cell lung cancer

**DOI:** 10.3389/fonc.2023.1162181

**Published:** 2023-05-05

**Authors:** Dongrui Zhang, Baohua Lu, Bowen Liang, Bo Li, Ziyu Wang, Meng Gu, Wei Jia, Yuanming Pan

**Affiliations:** ^1^ Department of Respiratory and Critical Care Medicine, Tianjin Chest Hospital, Tianjin, China; ^2^ Department of Oncology, Beijing Chest Hospital, Capital Medical University, Beijing Tuberculosis and Thoracic Tumor Research Institute, Beijing, China; ^3^ Department of Traditional Chinese Medicine, Beijing Chest Hospital, Capital Medical University, Beijing Tuberculosis and Thoracic Tumor Research Institute, Beijing, China; ^4^ Cancer Research Center, Beijing Chest Hospital, Capital Medical University, Beijing Tuberculosis and Thoracic Tumor Research Institute, Beijing, China

**Keywords:** neural network, deep learning, predictive model, clinical tool, small cell lung cancer

## Abstract

**Background:**

Small cell lung cancer (SCLC) is an aggressive and almost universally lethal neoplasm. There is no accurate predictive method for its prognosis. Artificial intelligence deep learning may bring new hope.

**Methods:**

By searching the Surveillance, Epidemiology, and End Results database (SEER), 21,093 patients’ clinical data were eventually included. Data were then divided into two groups (train dataset/test dataset). The train dataset (diagnosed in 2010–2014, N = 17,296) was utilized to conduct a deep learning survival model, validated by itself and the test dataset (diagnosed in 2015, N = 3,797) in parallel. According to clinical experience, age, sex, tumor site, T, N, M stage (7th American Joint Committee on Cancer TNM stage), tumor size, surgery, chemotherapy, radiotherapy, and history of malignancy were chosen as predictive clinical features. The C-index was the main indicator to evaluate model performance.

**Results:**

The predictive model had a 0.7181 C-index (95% confidence intervals, CIs, 0.7174–0.7187) in the train dataset and a 0.7208 C-index (95% CIs, 0.7202–0.7215) in the test dataset. These indicated that it had a reliable predictive value on OS for SCLC, so it was then packaged as a Windows software which is free for doctors, researchers, and patients to use.

**Conclusion:**

The interpretable deep learning survival predictive tool for small cell lung cancer developed by this study had a reliable predictive value on their overall survival. More biomarkers may help improve the prognostic predictive performance of small cell lung cancer.

## Background

In 2022, around 609,360 cancer deaths were projected to occur in the United States, including approximately 350 deaths per day from lung cancer, the leading cause of cancer death, according to the estimate from the American Cancer Society (1). Moreover, it is estimated that the cancer burden will be projected to double by 2050, of which lung cancer will be at the top of the list (2). Approximately, lung cancer can be classified into two subtypes, small cell lung cancer (SCLC) (accounting for 15% of the total) or non-small cell lung cancer (around 85% of the total). SCLC is an almost universally lethal neoplasm, with rapid growth, aggressive proliferation, and high rate of metastasis (3, 4). Although SCLC is usually sensitive to chemotherapy or radiotherapy, this fast-growing neuroendocrine lung cancer owns a <7% 5-year survival rate only (around 15%–30% in limited-stage and <1% in extensive-stage) (5–8). Frustratingly, there has been little progress in its treatment for nearly 30 years (7).

Predicting SCLC patients’ overall survival (OS) might offer some references to clinicians. The most common reference is the American Joint Committee on Cancer (AJCC) TNM stage. By evaluating patients’ stage, doctors can know their rough survival probabilities. Considering TNM only includes tumor size and lymph node and distant metastases, there are still some controversies in its accuracy. For example, Shuai Shi and colleagues found that the 8th edition AJCC TNM stage for non-small cell lung cancer was not applicable to lung cancer as a second primary malignancy (9). Salomon Tendler and colleagues even mentioned that single metastatic lesions (M1b) had a better prognosis compared with M1c, when validating the 8th TNM stage for SCLC in a retrospective material from Sweden (10). Ziqing Zeng also pointed out that patients owning the same stage and similar treatment might differ in survival (11, 12). Another choice was to use traditional statistical models to achieve predictive tasks. The Cox proportional hazard (CPH) model is a classical statistical method, widely used in clinical practice. The CPH model calculates the linear association between outcome and related factors and can give a patient’s survival probability by accumulating all risk factor scores. However, it can be too simplistic to assume that the association is linear, especially in a complex and realistic clinically scenario (13).

Given the limit from the TNM stage or CPH, clinical researchers turned their attention to deep learning. Deep learning networks usually include several layers, like input layers, hidden layers, and output layers. Each layer contains certain nodes, and nodes between adjacent layers will develop connections weighted by some parameters, which are refreshed by lots of epochs to simulate and study the sophisticated association between inputted variables (like clinical features) and outcome. Due to the above highlights, deep learning can discover the underlying laws between predictive variables and outcome, by learning the complex, non-linear relationships and representation levels, which usually shows more robust performance than statistical methods like CPH or a traditional machine learning algorithm (14, 15). As a subdiscipline of machine learning, deep learning was increasingly valued by clinicians after displaying its advantages especially in handling big data (16–20). In order to handle survival data, Jared L. Katzman suggested a modern Cox proportional hazards model based on the deep learning method, named DeepSurv, to better fit this realistic non-linear application (13). DeepSurv combined the advantages of CPH and deep learning algorithm and can simulate more complex relationships. Yunlang She noted that DeepSurv showed great potential in providing individual prognostic information and treatment recommendations in non-small cell lung cancer, with areas under the receiver operating curve (AUCs) scoring 0.739 and 0.742 in internal and external validations, respectively (21). Homoplastically, Quincy A. Hathaway and colleagues discovered that DeepSurv can leverage simple clinical features alone to accurately predict atherosclerotic cardiovascular disease risks and cardiovascular outcomes (22). John Adeoye and colleagues pointed out that DeepSurv can accurately predict the malignant-transformation-free survival of oral potentially malignant disorders. Therefore, using this deep learning survival algorithm to build a predictive tool for SCLC patients was suitable and needed.

All data used in this study came from the Surveillance, Epidemiology, and End Results database (SEER). It is an authoritative and almost broadest coverage of source for cancer statistics in the United States, managed by the National Cancer Institute (NCI). Many doctors and researchers have used it to obtain more detailed and realistic clinical data and gain more comprehensive understanding of tumors (1, 23, 24). We chose it to search data and conduct models, which might return reliable clinical information and a robustly predictive model.

In this study, we collected SCLC patients’ clinical data from SEER and chose age, sex, tumor site, T, N, M stage, tumor size, surgery, chemotherapy, radiotherapy, and history of malignancy to act as predictive clinical features, then first conducted an interpretable deep learning-based OS predictive model for SCLC. The model showed certain predictive accuracy, so we packaged and uploaded it. This tool is very friendly. Users only need to input the above clinical information and click Predict, and the software will automatically call the pretrained dense neural network to convert the clinical information into the survival curve of that patient. It is free for doctors, researchers, and patients to use.

## Methods

### Study design and data collection

**Figure 1:**
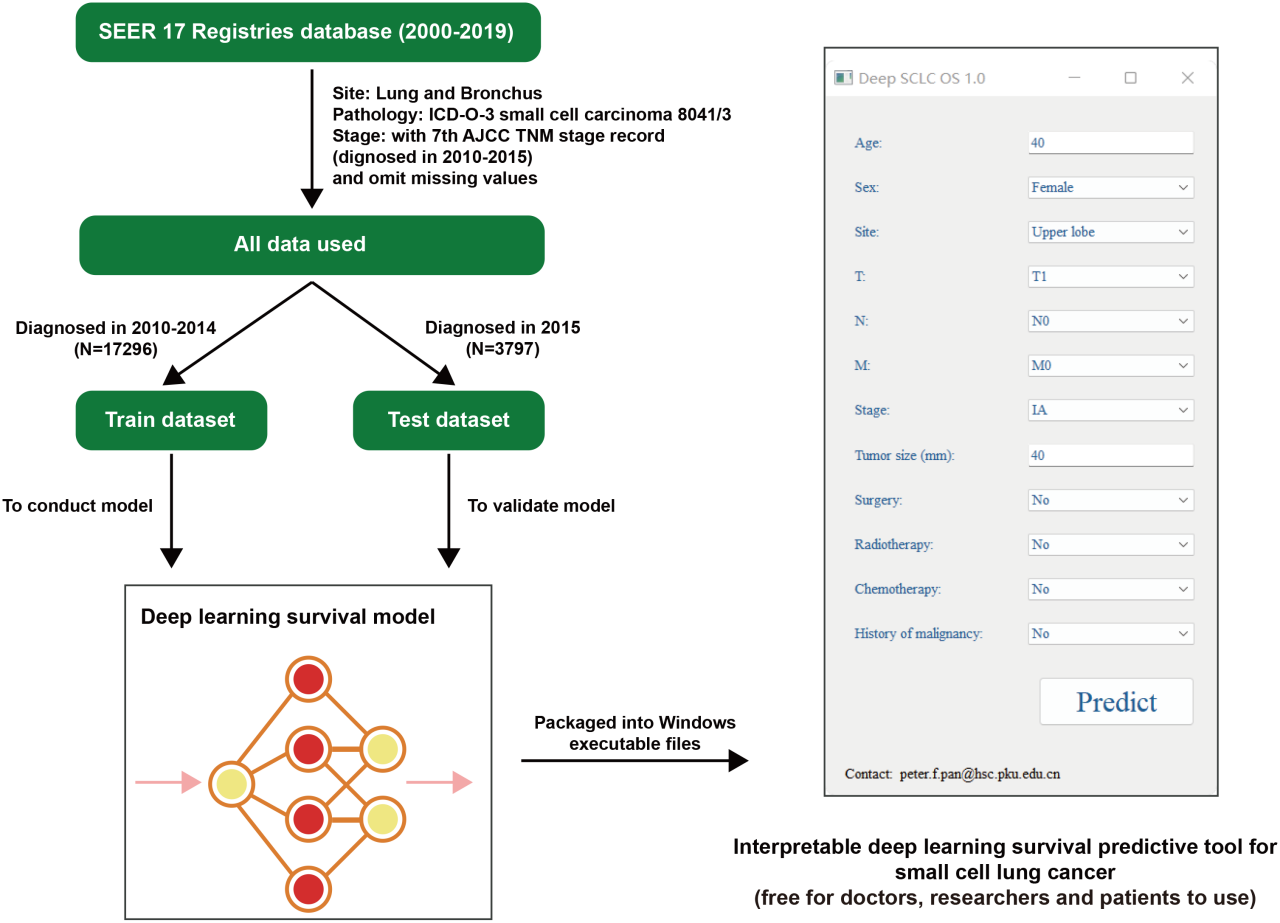
Flowchart of this research. SEER, the Surveillance, Epidemiology, and End Results database; AJCC, American Joint Committee on Cancer; SCLC, small cell lung cancer. OS, overall survival.

This study was designed as a retrospective cohort study. We browsed and searched SEER, downloading the 17 Registries database (2000–2019) which covers approximately 26.5% of the US population. By setting *Site: Lung and Bronchus* and *Pathology: ICD-O-3 Small cell carcinoma 8041/3*, we acquired the raw data. Considering that the follow-up cutoff date was December 31, 2019 (according to SEER’s official manual), and patients staged by the 8th AJCC TNM stage (mainly used from 2018) without sufficient duration of follow-up, we checked raw data to make sure its 7th AJCC TNM stage information (mainly patients diagnosed in 2010–2015) is intact. With missing values omitted, data with a sample size of 21,093 were eventually included by this study. Based on the diagnosed time, data were then divided into two groups (train dataset/test dataset), and the train dataset (diagnosed in 2010-2014) was utilized to conduct a deep learning survival model, validated by itself and the test dataset (diagnosed in 2015) in parallel (**Figure 1**).

### Predictive clinical features

According to clinical experience, age, sex, tumor site, T, N, M stage (7th AJCC TNM stage), tumor size, surgery, chemotherapy, radiotherapy, and history of malignancy were chosen as predictive clinical features. Age, more advanced stage (T, N, M stage), and bigger tumor size were classic risk factors of SCLC, so they were included (25, 26). Meanwhile, tumor sites might also influence clinical treatment (those in the main trachea cannot be easily removed, and tumors near large blood vessels or important organs would not be easily treated with radiotherapy). Therefore, we included tumor site and the most common therapy in SCLC (surgery, chemotherapy, and radiotherapy). Although there is no consensus on the effect of sex on cancer’s prognosis yet, considering the differences in living habits between different genders and the potential influence of hormones, we also included them like in previous studies (27, 28). Malignancy history was a risk factor for most cancers, which might indicate the underlying gene mutations carried by individuals and damage the patient’s health, increasing the occurrence of additional cancers, and thus it served as a prognostic indicator too (29).

Due to SEER record regulation, patients older than 100 years were still recorded as 100-year-olds. Also, survival time of less than 1 month was still documented as 1 month.

### Model conduction and evaluation

The DeepSurv algorithm, a CPH deep neural network and state-of-the-art survival method for modeling interactions between a patient’s covariates and treatment, was taken to complete this survival-predictive model for SCLC (13). This model had two hidden layers (16 and 8 nodes each) and batch normalization layers. Numeric clinical features (age and tumor size) were normalized (subtract the mean and divide by the standard deviation), and the test dataset was normalized too in compliance with the train dataset. The StandardScaler function from Python package sklearn was utilized to achieve the above process, and related parameters were saved in joblib file where we can use its dump function to store parameters and use the load function to call back then standardize data automatically when necessary.

Categorical clinical features (sex, tumor site, T, N, M, stage, surgery, chemotherapy, radiotherapy and history of malignancy) were converted to dummy variables (replace characters with numbers, such as replacing Female as 1). This procedure was accomplished manually. **Supplement Table 1** contained the complete numerical codes.

To avoid overfitting, early stopping function were turned on to end training timely, whereas dropout layers were added too to prevent certain nodes from extremely high weights.

The model was conducted only using the train dataset but validated by both the train dataset and test dataset. The C-index was the main indicator to evaluate model performance. The closer the C-index is to 1, the better the model’s performance is, and the closer the C-index is to 0.5, the worse the model’s performance is. A model with a C-index greater than 0.7–0.8 is considered to have a good predictive value, usually. In order to get the C-index and 95% confidence intervals (CIs), we used 1,000 times bootstrap in both datasets.

### Models packaged into tool

Given the model’s usability and convenience, we packaged it into a runnable Windows executable files (in an environment of Windows 11, 64-bit version). This deep learning survival predictive tool for SCLC was free for doctors, researchers, and patients to use.

### Statistical analysis

All analysis was completed with R software (https://www.r-project.org/). Non-normally distributed numeric data were analyzed by Wilcoxon test, whereas categorical data were compared by the chi-square test. A two-sided P less than 0.05 was considered statistically significant.

## Results

### Clinical features of patients

**Figure 2:**
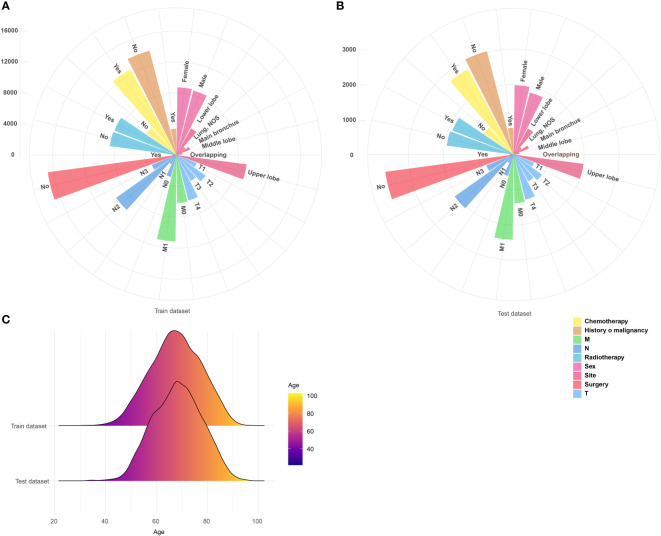
Visualized presentation of the overall categorical clinical features from the **(A)** train dataset and **(B)** test dataset. **(C)** The age profile of above datasets. NOS, not otherwise specified. Stage was evaluated according to the 7th American Joint Committee on Cancer TNM stage.

A total of 21,093 patients were eventually included by this study. The train dataset (diagnosed in 2010–2014, N = 17,296) and test dataset (diagnosed in 2015, N = 3,797) had similar clinical features in sex, tumor site, T, M, stage, tumor size, surgery, chemotherapy, radiotherapy, and history of malignancy, but there was a difference in age and N of two datasets (**Table 1**). **Figure 2** shows the presentation of the overall clinical features too, of which categorical clinical features were shown by the (A) train dataset and (B) test dataset and illustrates (C) the age profile of two datasets. The median follow-up time of both datasets was 8 months.

**Table 1:** The clinical features of SCLC patients.

	Train dataset	Test dataset	Statistical method	P value
	(N = 17,296)	(N = 3,797)		
	N (%)			
Age			Wilcoxon	<0.001***
Median (IQR)	68 (61, 75)	68 (61, 75)		
Sex			Chi-square	0.0761
Female	8,717 (50.4)	1,974 (51.99)		
Male	8,579 (49.6)	1,823 (48.01)		
Site			Chi-square	0.2382
Upper lobe	9,132 (52.8)	2,002 (52.73)		
Middle lobe	733 (4.24)	162 (4.27)		
Lower lobe	3,920 (22.66)	846 (22.28)		
Main bronchus	1,894 (10.95)	463 (12.19)		
Overlapping	225 (1.3)	45 (1.19)		
Lung, NOS	1,392 (8.05)	279 (7.35)		
T			Chi-square	0.3815
T1	19 (0.11)	1 (0.03)		
T1a	1,459 (8.44)	348 (9.17)		
T1b	1,327 (7.67)	275 (7.24)		
T2	55 (0.32)	8 (0.21)		
T2a	3,084 (17.83)	659 (17.36)		
T2b	1,477 (8.54)	316 (8.32)		
T3	3,822 (22.1)	866 (22.81)		
T4	6,053 (35)	1,324 (34.87)		
N			Chi-square	0.0102*
N0	2,922 (16.89)	630 (16.59)		
N1	1,384 (8)	270 (7.11)		
N2	9,544 (55.18)	2,057 (54.17)		
N3	3,446 (19.92)	840 (22.12)		
M			Chi-square	0.6119
M0	6,174 (35.7)	1,379 (36.32)		
M1	127 (0.73)	32 (0.84)		
M1a	1,802 (10.42)	374 (9.85)		
M1b	9,193 (53.15)	2,012 (52.99)		
Stage			Chi-square	0.3528
IA	552 (3.19)	131 (3.45)		
IB	372 (2.15)	66 (1.74)		
II	2 (0.01)	1 (0.03)		
IIA	473 (2.73)	91 (2.4)		
IIB	270 (1.56)	59 (1.55)		
IIIA	2,730 (15.78)	643 (16.93)		
IIIB	1,775 (10.26)	388 (10.22)		
IV	11,122 (64.3)	2,418 (63.68)		
Tumor size (mm)			Wilcoxon	0.1282
Median (IQR)	46 (28, 70)	48 (28, 71)		
Surgery			Chi-square	0.4981
No	16,754 (96.87)	3,686 (97.08)		
Yes	542 (3.13)	111 (2.92)		
Radiotherapy			Chi-square	0.6186
No	8,687 (50.23)	1,924 (50.67)		
Yes	8,609 (49.77)	1,873 (49.33)		
Chemotherapy			Chi-square	0.9197
No	4,765 (27.55)	1,043 (27.47)		
Yes	12,531 (72.45)	2,754 (72.53)		
Survival time			Wilcoxon	0.3260
Median (IQR)	8 (2, 16)	8 (2, 16)		
History of malignancy			Chi-square	0.5953
No	13,886 (80.28)	3,034 (79.91)		
Yes	3,410 (19.72)	763 (20.09)		

IQR, interquartile range; NOS, not otherwise specified. Stage was evaluated according to the 7th American Joint Committee on Cancer TNM stage. The follow-up cutoff date was December 31, 2019 (according to SEER’s official manual).

### Model training and performance

As we talked, the train dataset was used to conduct the model, which was then validated by both the train dataset and test dataset. After 294 epochs, early stopping function terminated the model training automatically. The training curves are shown in **Supplementary Figure 1**.

Finally, this model scored 0.7181 in the train dataset and 0.7210 in the test dataset on the C-index. After 1,000 times bootstrap, the predictive model had a 0.7181 C-index (95% CIs, 0.7174–0.7187) in the train dataset and 0.7208 C-index (95% CIs, 0.7202–0.7215) in the test dataset. The above indicated that the model had a reliable predictive value on OS for SCLC patients (**Table 2**).

**Table 2:** The model performance in the SCLC database.

	Train dataset		Test dataset	
	Mean	95% CIs	Mean	95% CIs
C-index	0.7181	–	0.7210	–
C-index from bootstrap	0.7181	0.7174–0.7187	0.7208	0.7202–0.7215

1,000 times bootstrap was used to obtain 95% CIs. CIs, confidence intervals.

**Figure 3:**
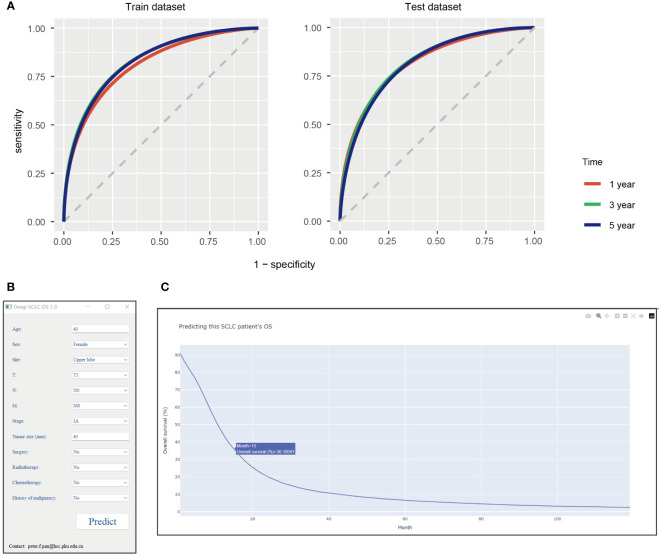
**(A)** The receiver operating curve of this model at 1, 3, and 5 years. Interpretable deep learning survival predictive tool for small cell lung cancer: **(B)** tool interface, **(C)** screenshot of the results when tool running. SCLC, small cell lung cancer; OS, overall survival.

We also tested the model’s performance at 1, 3, and 5 years. The model showed 0.8087 AUC, 0.7518 specificity, 0.7123 sensitivity, and 0.7254 accuracy at 1 year in the train dataset, and 0.8175 AUC, 0.7549 specificity, 0.7241 sensitivity, and 0.7341 accuracy at 1 year in the test dataset (**Table 3**). Meanwhile, the model exhibited 0.8300 AUC, 0.7587 specificity, 0.7417 sensitivity, and 0.7435 accuracy at 3 years in the train dataset and 0. 8228 AUC, 0.7419 specificity, 0.7456 sensitivity, and 0.7452 accuracy at 3 years in the test dataset (**Table 3**). Moreover, it had 0.8274 AUC, 0.7499 specificity, 0.7456 sensitivity, and 0.7459 accuracy at 5 years in the train dataset and 0.8161 AUC, 0.7247 specificity, 0.7515 sensitivity, and 0.7492 accuracy at 5 years in the test dataset (**Table 3**). The receiver operating curve also demonstrated that this model had a satisfactory predictive value (**Figure 3A**).

**Table 3:** Model performance at 1, 3, and 5 years.

	Train dataset	Test dataset
1 year		
AUC	0.8087	0.8175
AUC 95% CIs	0.8021–0.8157	0.8039–0.8329
Specificity	0.7518	0.7549
Sensitivity	0.7123	0.7241
Accuracy	0.7254	0.7341
3 years		
AUC	0.8300	0.8228
AUC 95% CIs	0.8208–0.8395	0.8019–0.8439
Specificity	0.7587	0.7419
Sensitivity	0.7417	0.7456
Accuracy	0.7435	0.7452
5 years		
AUC	0.8274	0.8161
AUC 95% CIs	0.8152–0.8393	0.7923–0.8414
Specificity	0.7499	0.7247
Sensitivity	0.7456	0.7515
Accuracy	0.7459	0.7492

AUC, area under the receiver operating characteristic curve; CIs, confidence intervals. 1000 times bootstrap was used to obtain 95% CIs.

The detailed hyperparameters of this model are shown in **Supplement Figure 2**.

### Model wrapped into the tool

Then, we wrapped this deep learning model into Windows executable files. This tool can be installed after launching **Supplementary File 1**. When inputting an SCLC patient relevant clinical feature, users can click the “Predict” button to start up the deep learning model for calculating (**Figure 3B**). After a minute, a website will be open in the system default web browser (**Figure 3C**). This website shows the predictive survival curve, as the x-axis represents months and the y-axis is the predictive OS probability of this patient. This survival curve is interactable, for specific values can be displayed when mouse hovering and be able to zoom in or out. It’s free for doctors, researchers and patients to use.

## Discussion

As a subtype of lung cancer, SCLC arises in peribronchial locations and infiltrates the bronchial submucosa, with many new cases diagnosed every year (30). One to five persons per 10,000 people can be diagnosed with SCLC in the European community, more prevalent in men than women (31–33). As for the treatment of SCLC, some scholars have pessimistically concluded that there has been no virtual progress in the treatment of SCLC for 30 years (34).Considering that only a tiny subset of individuals with SCLC can get surgery, the classical treatment regimen for them is platinum-based chemotherapy (35). Unfortunately, although showing a robust response to initial therapy, almost all SCLC patients recur and are resistant to further therapy, with a median OS of 10 months after first-line chemotherapy (36, 37). Similarly, after receiving therapy with first- or second-generation EGFR tyrosine kinase inhibitors for 9 to 15 months, the majority of SCLC patients inevitably develop acquired resistance through a variety of mechanisms (38–40). Immune checkpoint inhibitors (ICIs) like programmed death-ligand 1 (PD-L1) were exciting new therapeutic agents for SCLC in recent years. In the IMPower133 trial, SCLC patients receiving atezolizumab plus carboplatin and etoposide had 12.3 months of median OS (hazard ratio, HR, 0.76; 95% CIs, 0.60–0.95; P = 0.0154), compared with 10.3 months from the placebo, atezolizumab, and carboplatin group (41). In the CASPIAN trial, durvalumab plus platinum-etoposide patients had a longer OS around 13.0 median months (HR 0.73; 95% CIs 0.59–0.91; P = 0.0047) compared to the platinum-etoposide group (around 10.3 median OS) (42). However, in KEYNOTE-604, pembrolizumab plus etoposide and platinum did not improve SCLC patient OS significantly (43). Meanwhile, it must be mentioned that the expression of PD-L1 was less common in SCLC patients according to reports. The PD-L1 expression level in immune cells was <1% in almost half of the cases (68/137), whereas it was <1% in nearly all samples (129/137) of cancer cells in IMPower133 (41, 44). Therefore, despite the fact that ICIs plus standard chemotherapy might prolong SCLC patients’ OS, there is still a long way to go due to the following: the limited OS improvement, only a small number of beneficial patients, and the lack of predictive biomarkers (45–47). Hence, predicting the OS of SCLC patients can help guide clinical decisions by taking into account the side effects and unfavorable consequences of chemotherapy, targeted therapy, and immunotherapy.

In respect of the management of SCLC patients, a used staging system in the past was the Veterans Administration Lung Study Group (VALSG), grouping SCLC into limited-stage or extensive-stage (48). However, more researchers appealed to use the AJCC TNM stage instead to obtain more benefit in defining optimal treatment strategies these years (10, 35, 49, 50). As we mentioned earlier, there are still some controversies in the TNM staging system’s accuracy for it only includes tumor size, lymph node, and distant metastasis. Previous researchers have tried to develop new models to predict SCLC’s survival. Shidan Wang et al. used age, gender, race, ethnicity, Charlson/Deyo score, TNM stage, treatment type, and laterality to act as predictive variables to develop an SCLC predictive nomogram based on CPH and The National Cancer Database, with a concordance index of 0.722 (51). However, the Charlson/Deyo score is not widely used in some countries, which might limit the application of this model. Hui Pan and colleagues utilized clinical features and CPH to predict SCLC patients’ OS, the model scoring a 0.68 concordance index in the primary cohort and 0.66 in the validation cohort (52). Zeng et al. established a prognostic model for the survival of resected limited-stage SCLC, with a performance 0.722–0.746 in the train cohort and 0.693–0.816 in the validation cohort (53). Simultaneously, some researchers attempted to use biomarkers to predict SCLC’s clinical outcome. Minlin Jiang and colleagues built a FOXP3-based immune risk model to predict I–III stage SCLC’s recurrence, and the model scored 0.656–0.737 AUC in the train cohort and 0.608–0.714 in the validation cohort (54). Xie et al. used peripheral blood markers and CPH to develop a model predicting SCLC’s prognosis with a 0.73 concordance index (55). Shicheng Feng and colleagues identified six novel prognostic gene signatures, which scored 0.825 AUC in SCLC’s prognostic prediction (56). Biomarkers appeared to improve the predictive performance of prognostic models for SCLC, but these materials were not readily available, especially in SCLC for the limited surgical chance.

Deep learning was a novel machine learning algorithm that has been transplanted to the medical field with attention in recent years. It has been demonstrated that it can discriminate high-risk smokers for lung cancer screening computed tomography, Coronavirus disease 2019, fibrotic lung disease, histopathological whole slide images, and so on (57–60). Yunlang She and colleagues have built a deep learning survival model to predict non-small cell lung cancer survival (21). This model had a 0.739 C statistic and was visualized by a user-friendly graphic interface then. However, SCLC still lacks such model to help in clinical decision making. In this study, we pioneered to conduct an interpretable deep learning survival predictive model for SCLC and packaged it into an accessible tool. This model had a reliable predictive performance, with a 0.7181 C-index in the train dataset and 0.7208 in the test dataset.

The median age of SCLC patients in our data was 68 years, similar to previous studies (61–63). Older SCLC patients are thought to have a poorer prognosis, especially older than 60 or 70 years (3, 25, 26). Interestingly, in our tests of this deep learning model, we found that it thought age had limited weight and little influence on OS in most cases. The potential cause might be that we considered age to be a numeric rather than a categorical variable. As a result, an age difference of decades has no significant influence on prognosis. Although it is very common to use age truncation as categorical data in statistical analysis, we still insist on treating it as a continuous variable this time, which is also the most common way of processing in deep learning. Meanwhile, the proportion of male and female patients was approximately equal in our research, as the study by Lin Gao (61), but different from that of Hidefumi Takei or Kosei Doshita (significantly more for men) (63, 64). This difference might be ethnographic between Asians and Americans, but more research was needed to confirm. Patients with SCLC were more often diagnosed with advanced (stages III–IV) in our observation, which was the same with earlier studies (61, 63, 65, 66) but different from the data from Japan between 2004 and 2010 (64). The discrepancies might derive from recent advances in screening tools.

There were still some limitations in this study. This deep learning survival model might need to be validated further by Asian population data. Certain personal history like smoking status, parental cancer diagnosis, or gene-based biomarker might be an implication for patients’ survival but might not be included this time.

To make a long story short, we first built an interpretable deep learning survival predictive tool for SCLC using 21,093 patients’ data. This tool had a reliable predictive value on OS for SCLC with a 0.7181 C-index (95% CIs, 0.7174–0.7187) in the train dataset and a 0.7208 C-index (95% CIs, 0.7202–0.7215) in the test dataset.

## Conclusion

An interpretable deep learning survival predictive tool for small cell lung cancer developed by this study had a reliable predictive value on their overall survival. More biomarkers may help improve the prognostic predictive performance of small cell lung cancer.

## Data availability statement

The original contributions presented in the study are included in the article/**Supplementary Material**. Further inquiries can be directed to the corresponding authors.

## Ethics statement

This study has been approved by the Ethics Committee of Tianjin Chest Hospital and individual consent for this retrospective analysis was waived. Written informed consent for participation was not required for this study in accordance with the national legislation and the institutional requirements.

## Author contributions

(I) Conception and design: DZ and WJ. (II) administrative support: DZ and YP. (III) provision of study materials or patients: BL, BHL, YP, ZW, MG and BWL. (IV) collection and assembly of data: BL, BHL, YP, ZW, MG and BWL. (V) data analysis and interpretation: DZ and YP. All authors contributed to the article and approved the submitted version.

## Supplementary material

Supplementary Figure 1 The training curves of deep learning survival predictive model for SCLC.

Supplementary Figure 2 The detailed hyper-parameters of this model.

Supplementary File 1 Installation file of interpretable deep learning survival predictive tool for SCLC. (https://drive.google.com/file/d/1yjdh-mSkqTOEPpsV21t8hd4drjimmY3V/view?usp=share_link).

Supplement File 2 The raw data of this study.

Supplementary Table 1 Numerical codes of categorical variables. NOS, not otherwise specified.
