# Supplementary figures and images for: Interpretable deep learning survival predictive tool for small cell lung cancer
Source: Front Oncol. 2023 May 5;13:1162181. doi: 10.3389/fonc.2023.1162181 (PMC10196231; doi:10.3389/fonc.2023.1162181)

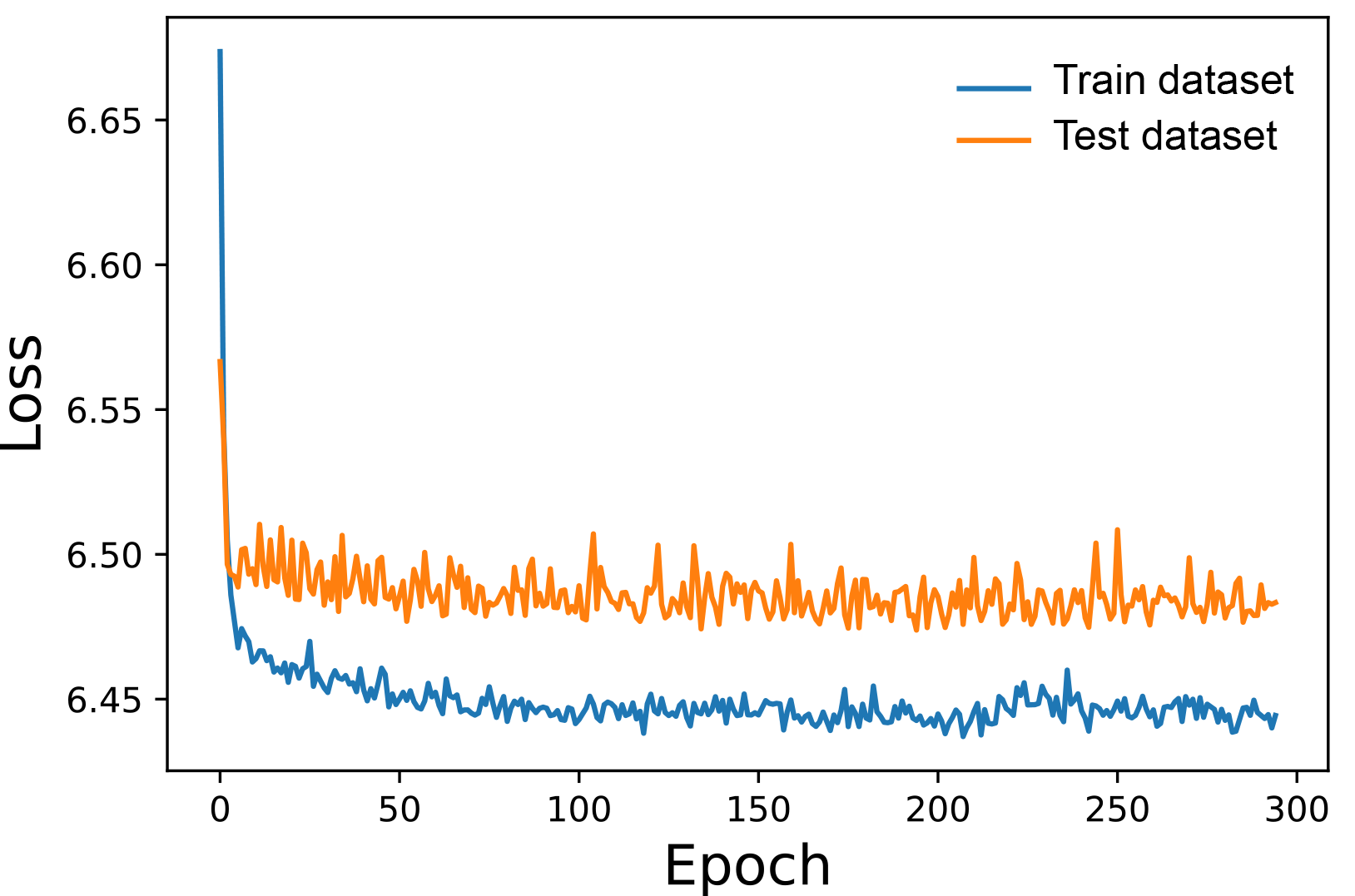

Supplement: Supplementary Figure 1 — The training curves of deep learning survival predictive model for SCLC. [file Image_1.tif]
