# Supplementary material for: Interpretable deep learning survival predictive tool for small cell lung cancer
Source: Front Oncol. 2023 May 5;13:1162181. doi: 10.3389/fonc.2023.1162181 (PMC10196231; doi:10.3389/fonc.2023.1162181)
Supplement: Supplementary Figure 2 — The detailed hyper-parameters of this model. [file Image_2.pdf]

Hyper-parameters

OrderedDict([('net.0.linear.weight', tensor([[-6.4332e-01, 1.3462e-01, -6.9734e-01, -2.155
5e-01, 4.1805e-02,
-2.7495e-01, -5.6994e-01, -7.9306e-02, 4.0744e+00, 2.2376e+00,
2.5589e+00, -4.3109e-01],
[2.9291e-01, -4.2745e-02, 6.0576e-01, 2.3374e-01, 1.5103e-01,
2.4792e-01, 4.8991e-01, 5.6756e-02, -2.7695e+00, -2.3963e+00,
-1.6811e+00, 5.0877e-01],
[-3.4341e-01, -9.4389e-02, -3.8128e-01, -1.6443e-03, -6.0993e-02,
-6.5324e-02, -4.5558e-01, -7.2538e-02, 1.5756e+00, 5.1375e-01,
2.5935e+00, 1.9583e-01],
[-6.3694e-01, -1.3277e+00, -6.3058e-02, 2.0232e-02, 7.7614e-02,
-2.1875e-01, -5.6302e-01, -7.7263e-02, 2.3717e+00, 4.8443e-01,
3.5615e+00, 3.9231e-01],
[1.5168e-01, -1.4059e-01, 1.1810e-01, 5.4498e-02, -3.3226e-01,
-6.6174e-01, 2.0687e-01, -3.9788e-01, -5.5598e-01, 6.7530e-01,
-6.7516e-01, -1.0493e+00],
[7.7874e-01, -2.0355e-01, 7.7204e-01, -2.6032e-02, 9.6580e-03,
6.4851e-01, 6.8044e-01, 6.8282e-01, 2.7543e-01, -8.4528e-01,
-3.5437e+00, -1.6646e-01],
[-3.0909e-01, 1.9614e-01, -2.4327e-01, -5.6191e-01, -2.1284e-01,
5.4548e-01, -2.5755e-03, -6.2653e-01, 1.3812e-01, -4.8637e-01,
7.6415e-02, 1.2531e-01],
[8.0548e-02, 2.1411e-01, -1.6321e-01, -2.5353e-01, -6.7179e-01,
-7.7167e-01, 8.2236e-02, -3.9719e-01, -1.2591e+00, -1.4009e+00,
-5.0273e+00, -7.4244e-01],
[-4.8292e-01, -5.7996e-02, 5.6026e-01, -4.5560e-01, -1.2336e-01,
-7.3886e-01, -2.0391e-02, -5.4512e-01, 2.9093e-01, -1.7181e-01,
-5.6517e-01, -2.4574e-01],
[3.7397e-01, -3.1499e-02, 1.0486e+00, 4.5113e-02, 6.4808e-02,
5.3358e-01, 3.9076e-01, 5.9744e-01, -5.0624e-01, -2.1874e+00,

-2.1153e+00, 7.6047e-01],
[-3.9527e-01, 1.0801e-01, 7.8480e-04, -6.6551e-01, -4.5677e-01,
-1.7362e-01, -3.1919e-01, 2.3221e-01, -6.3229e-01, 1.2709e-01,
-4.1659e-01, 2.4819e-02],
[-4.3961e-01, 1.3046e-02, -2.5603e-01, 1.8493e-01, -1.1611e-01,
-5.3697e-02, -4.7376e-01, -3.3193e-01, 2.5793e+00, 9.3930e-01,
3.6328e+00, 2.5699e-01],
[-1.3762e+00, -1.7137e+00, 3.2583e-03, 1.4022e-01, 1.2182e-01,
-5.7163e-01, -2.0731e+00, -2.1431e-01, 2.7888e+00, 3.4030e+00,
-2.5050e+00, -4.9993e-01],
[-2.4118e-01, 1.8422e-01, -2.5894e-01, -6.7600e-01, -4.5020e-01,
-6.0479e-01, -1.0944e-01, -3.2805e-01, 2.0265e-01, 1.7403e-01,
1.5503e-01, -4.1809e-01],
[-5.2318e-01, -1.1993e-01, -4.5005e-01, -7.8448e-02, -3.1059e-02,
-1.3617e-01, -4.1902e-01, -1.7080e-01, 3.2724e+00, 9.5756e-01,
2.9957e+00, 3.3714e-01],
[5.4199e-01, 1.4462e-01, 1.1761e-01, -3.4361e-02, -3.9666e-02,
6.5902e-01, 2.1784e+00, -1.6721e-02, 7.8382e-01, -7.4535e-01,
-5.9021e+00, -1.2162e+00]])), ('net.0.linear.bias', tensor([4.9951e-01, 7.1408
e+00, 6.5823e-01, -1.0001e-01, -3.2195e-03,
2.0494e+00, -2.1113e-01, 4.3192e-01, -1.0993e-01, 2.3701e+00,
1.1160e-01, 6.7609e-01, 9.5785e-01, -2.5326e-01, 5.4946e-01,
-1.3544e-01])), ('net.0.batch_norm.weight', tensor([0.7805, 0.8051, 0.7581, 0.94
05, 1.3661, 0.6370, 1.0000, 0.8938, 1.3741,
0.6751, 1.0000, 0.9025, 0.8194, 1.0000, 0.7117, 1.5072])), ('net.0.batch_norm.
bias', tensor([-0.0682, 0.3604, 0.0099, 0.2012, 0.1899, -0.0203, -0.0675, 0.0114,
0.0723, 0.0416, 0.1353, 0.0588, -0.0198, 0.0858, -0.0439, 0.3738])), ('n
et.0.batch_norm.running_mean', tensor([7.0938e+00, 3.7340e+00, 3.8941e+00, 5.8492e+0
0, 5.6052e-45, 4.8026e+00,
0.0000e+00, 8.0421e-03, 5.6052e-45, 4.5814e+00, 0.0000e+00, 6.8016e+00,

7.9071e-01, 0.0000e+00, 7.0860e+00, 4.3279e-01])), ('net.0.batch_norm.running_var', tensor([7.2405e+00, 4.8379e+00, 3.3139e+00, 6.7689e+00, 1.4854e-43, 8.7564e+00,

1.4854e-43, 1.8896e-02, 1.4854e-43, 6.4715e+00, 1.4854e-43, 6.3013e+00,

2.4554e+00, 1.4854e-43, 5.0232e+00, 1.0423e+00])), ('net.0.batch_norm.num_batches_tracked', tensor(936)), ('net.1.linear.weight', tensor([[0.4299, -0.2606, 0.4662, 0.3070, -0.5880, -0.7117, 0.3058, -0.4652,

-0.5444, -0.3248, -0.1196, 0.1000, 0.4518, 0.5855, 0.3140, -0.5592],

[0.2999, -0.2979, 0.3012, 0.3234, -0.2282, -0.2261, 0.3375, -0.4833,

0.4082, -0.1760, 0.3665, 0.4161, 0.4816, -0.3067, 0.3970, -0.7162],

[-0.0781, -0.1845, -1.0297, -2.1930, 1.0561, 0.5937, 0.1618, 0.8531,

-0.0607, 0.0983, 0.3907, -1.0041, -0.1446, -0.0203, -1.1895, 0.3278],

[0.3287, -0.3153, 0.5078, 0.4281, -0.0922, -0.5508, 0.1133, -0.4536,

-0.4022, -0.2281, -0.3816, 0.4051, 0.3484, 0.4014, 0.2964, -0.6058],

[0.2410, -0.0618, 0.2841, 0.2231, 0.5264, -0.3734, 0.8653, -0.4761,

0.1631, -0.0837, 0.9286, 0.3375, 0.1724, -0.6918, 0.3802, -0.7917],

[0.7509, -1.3316, -0.0302, -0.2564, 0.5631, -0.4372, 0.2439, 0.0285,

0.5687, -1.1661, 0.2383, -0.0081, 0.0510, 0.4700, -0.0214, -0.3467],

[-0.6596, 0.3645, -0.4124, -0.1188, 0.0976, 0.1951, 0.8348, -0.1105,

0.6643, 0.3416, 0.9820, -0.3214, -1.0908, -1.2739, -0.6376, 0.3575],

[0.1332, -0.3611, 0.3191, 0.2065, 0.3859, -0.6342, -0.4449, -0.4689,

-0.1686, -0.0768, -0.3300, 0.7641, 0.1753, 0.2553, 0.3849, -0.8701]])), ('net.1.linear.bias', tensor([5.0838, 3.3894, 1.5381, 4.2225, 3.8561, 1.2405, 2.7729, 4.3783])), ('net.1.batch_norm.weight', tensor([0.4758, 0.4807, 0.4879, 0.4986, 0.4593, 0.4906, 0.5264, 0.4460])), ('net.1.batch_norm.bias', tensor([0.0502, -0.0960, 0.0599, -0.0018, 0.0168, -0.0763, 0.0319, 0.0893])), ('net.1.batch_norm.running_mean', tensor([4.8164, 3.3895, 2.7368, 4.1321, 3.8802, 1.3823, 3.5267, 4.3672])), ('net.1.batch_norm.running_var', tensor([7.3706, 5.1579, 15.9035, 7.0116, 4.4498, 2.8126, 7.3903, 7.0557])), ('net.1.batch_norm.num_batches_tracked', tensor(936)), ('net.2.weight', tensor([[-0.2872, -0.1872, 0.2205, -0.2390, -0.2465, -0.2224, 0.2533, -0.2290]]))])
