# Supplementary material for: Interpretable deep learning survival predictive tool for small cell lung cancer
Source: Front Oncol. 2023 May 5;13:1162181. doi: 10.3389/fonc.2023.1162181 (PMC10196231; doi:10.3389/fonc.2023.1162181)
Supplement: Supplementary Table 1 — Numerical codes of categorical variables. NOS, not otherwise specified. [file Table_1.docx]

| Supplement Table 1. Numerical codes of categorical variables. | |
| --- | --- |
| Clinical feature | Code |
| Sex |  |
| Female | 1 |
| Male | 2 |
| Site |  |
| Upper lobe | 1 |
| Middle lobe | 2 |
| Lower lobe | 3 |
| Main bronchus | 4 |
| Overlapping | 5 |
| Lung, NOS | 6 |
| T |  |
| T1 | 1 |
| T1a | 2 |
| T1b | 3 |
| T2 | 4 |
| T2a | 5 |
| T2b | 6 |
| T3 | 7 |
| T4 | 8 |
| N |  |
| N0 | 1 |
| N1 | 2 |
| N2 | 3 |
| N3 | 4 |
| M |  |
| M0 | 1 |
| M1 | 2 |
| M1a | 3 |
| M1b | 4 |
| Stage |  |
| IA | 1 |
| IB | 2 |
| II | 3 |
| IIA | 4 |
| IIB | 5 |
| IIIA | 6 |
| IIIB | 7 |
| IV | 8 |
| Surgery |  |
| No | 1 |
| Yes | 2 |
| Radiotherapy |  |
| No | 1 |
| Yes | 2 |
| Chemotherapy |  |
| No | 1 |
| Yes | 2 |
| History of malignancy | |
| No | 1 |
| Yes | 2 |
| NOS, not otherwise specified. | |
